# Smartphone-Based Platform for Secure Multi-Hop Message Dissemination in VANETs

**DOI:** 10.3390/s20020330

**Published:** 2020-01-07

**Authors:** Hiram Galeana-Zapién, Miguel Morales-Sandoval, Carlos A. Leyva-Vázquez, Javier Rubio-Loyola

**Affiliations:** Cinvestav Tamaulipas, Ciudad Victoria C.P. 87130, Mexico; mmorales@tamps.cinvestav.mx (M.M.-S.); cleyva@tamps.cinvestav.mx (C.A.L.-V.); jrubio@tamps.cinvestav.mx (J.R.-L.)

**Keywords:** dissemination protocols, global positioning system (GPS), mobile platform, security, vehicular ad-hoc

## Abstract

Vehicular ad-hoc Networks (VANETs) are recognized as a cornerstone of Intelligent Transportation Systems (ITS) to enable the exchange of information among vehicles, which is crucial for the provision of safety-related and entertainment applications. However, practical useful realizations of VANETs are still missing, mainly because of the elevated costs and the lack of a final standardization. In this regard, the feasibility of using smartphones as nodes in VANETs has been explored focusing on small-scale deployments to mainly validate single-hop communication capabilities. Moreover, existing smartphone-based platforms do not consider two crucial requirements in VANETs, namely, multi-hop communication and the provision of security services in the message dissemination process. Furthermore, the problem of securing message dissemination in VANETs is generally analyzed through simulation tools, while performance evaluations on smart devices have not been reported so far. In this paper, we aim to fill this void by designing a fully on-device platform for secure multi-hop message dissemination. We address the multi-hop nature of message dissemination in VANETs by integrating a location-based protocol that enables the selection of relay nodes and retransmissions criteria. As a main distinction, the platform incorporates a novel certificateless cryptographic scheme for ensuring data integrity and nodes’ authentication, suitable for VANETs lacking of infrastructure.

## 1. Introduction

As population densities increase in large cities around the world, the need of novel vehicular traffic management solutions for assuring safety while maintaining congestion at acceptable levels has become more evident. Intelligent transportation systems (ITS) are expected to fill this need, relieving congestion and improving safety [1]. This is achieved by integrating advanced wireless and wireline communication, sensor and processing technologies into transportation systems, and into vehicles themselves, in order to develop a wide range of applications (e.g., crash prevention and safety, freeway management, etc.). Such in-vehicle communication and computation capabilities are expected to enable vehicles to autonomously cooperate among themselves to share information about their surrounding [2]. Specifically, vehicular ad-hoc networks (VANETs) are seen as the appropriate communication technology to wirelessly disseminate information among moving entities. This is carried out by making use of the wireless communication channel as vehicles will be equipped with an on-board unit (OBU) that performs transmissions/receptions to/from nodes in a given zone of relevance. Depending on the communication paradigm, a vehicle can communicate to another vehicle, to a road side unit, or to a pedestrian handling a portable device, assuming a vehicle-to-vehicle (V2V), vehicle-to-infrastructure (V2I), vehicle-to-pedestrian (V2P), respectively. In this regard, data exchange through V2V links is an attractive approach to disseminate safety information among vehicles by means of multi-hop message dissemination protocols that operate on the network layer. In fact, this infrastructure-less communication paradigm is expected to address around 80% of precrash scenarios involving unimpaired drivers [3].

In this context, VANETs have been attracting attention in wireless communication and automotive industries in the last decade. In order to be practical and due to the high mobility of vehicles and the scarce deployment of VANETs’ infrastructure, WSNs (Wireless Sensor Networks) have been integrated in VANETs to form a hybrid vehicular environment which ensure a permanent connectivity between vehicles and favor timely detection of dangerous road conditions. Under this Hybrid Sensor and Vehicular Networks (HSVNs) [4], sensor nodes are deployed along the two sides of a road to assist VANET and provide better performance. Despite these trends and advances, VANETs market penetration has been low due to factors like implementation costs or user distrust, as has happened with other technologies like automated vehicles [5]. Such a low adoption rate of VANETs significantly contrasts with disruptive technologies like smartphones. In what follows, we analyze the recent trends and motivation of using smart devices in VANET scenarios to enable inter-vehicle communications, which is the main focus of this work.

### 1.1. Smartphones in VANETs

Nowadays, smart devices constitute the cornerstone of context-aware sensing applications on domains like m-health, smart-home, transportation, location-based services, etc. [6]. In such applications, smart devices could perform data sensing, pre-processing, feature extraction, classification, and processing. In this regard, the adoption of mobile edge computing solutions aims to take advantage of the increasing computing power of smartphones. While this paradigm shift is promoting the deployment of on-device processing capabilities in real-world mobile sensing systems, it remains a research challenge the efficient use of energy in constrained devices like smartphones. In vehicular networking, there are strong arguments that support the idea that the smartphone can become the platform enabling a fast realization of safety-related applications. First, inter-vehicular communications strongly depend on the successful development and market penetration of low-cost OBUs aligned to a wireless communication standard (In VANETs, the IEEE 802.11p standard is the most prominent option to enable inter-vehicular message dissemination). While the low-cost objective can be achieved by adopting off-the-shelf consumer electronics hardware and open-source software [7], we argue that backward compatibility (with old vehicles) should also be guaranteed. Hence, we believe that the adoption and the spread in large scale scenarios of OBUs is not expected to take place in the near future. Second, instead of the design of a fully specific device like OBUs, it is expected that smartphones play a key role in future vehicular networking as they provide a complete set of embedded sensors (GPS, accelerometer, etc.), computation and communication capabilities that enable and favor the deployment of VANET applications. Third, the adoption of smartphone-based platforms are likely to boost the development of vehicular applications as these devices are already widely used by the population [8,9]. This would allow to quickly realize safety applications in VANETs by exploiting their already large commercial location-based and mobility-based applications (e.g., Google Maps, WAZE, etc.) [10].

Due to this remarkable trend, the interest on the integration of smartphones into vehicles is gaining momentum in the academia, car manufacturers, phone companies, and infotainment system manufacturers, among others. For instance, smartphone-centric car connectivity solutions enhanced with V2I communications are the focus of the Car Connectivity Consortium (CCC). Recently, different research efforts have proposed the design of smartphone-based platforms to handle message dissemination in VANETs. On the one hand, the few existing proposals have been mainly focused on evaluating the feasibility of using smart devices to share information between two vehicles (i.e., single-hop V2V communication). On the other hand, however, the design of such mobile platforms do not consider two crucial aspects: (a) the provision of security services in the dissemination process, and (b) the feasibility of achieving the multi-hop dissemination process.

### 1.2. Related Work

The adoption of smartphones for the design of safety-related applications in VANETs is quite recent. Apart from initial efforts aimed at demonstrating the feasibility of using smartphones for vehicular networking purposes in simple communication scenarios [10,11,12,13], herein we concentrate on mobile platforms that have been proposed to perform experimental evaluation of message exchange through V2I and/or V2V communication paradigms in VANETs.

In [14], the authors deal with the design of a custom on-board unit based on a Raspberry Pi hardware module that manages 3G and Wi-Fi connections to enable applications running on users’ smartphones to select a network communication interface. In particular, applications interact with the hardware module through a REST API (application programming interface). The proposed platform is validated using an application to stream video from a vehicle to another overtaking vehicle, and mainly concentrating on transmission delay and throughput in the experimental scenarios. A similar approach was followed in [15], where an Arduino hardware collects vehicle statistics (e.g., engine’s temperature and revolutions, location) which are then processed and transmitted by a smartphone to a remote storage entity via Wi-Fi or 3G wireless interfaces for traffic analysis purposes. Notice that both ref. [14] and [15] mainly concentrate on data acquisition and processing from either a specific application or sensors attached to the custom hardware. Also, both proposals use the smartphone to communicate with an infrastructure and they do not provide any form of security mechanisms for such message exchange in the considered V2V and V2I communications, respectively. In [16], location information is collected from the native GPS sensor on a smartphone and disseminated in the vehicular network using Wi-Fi Direct. That approach follows a broadcast approach for message exchange between two surrounding entities (single-hop communication) but it does not support multi-hop message dissemination, which is mandatory to provide larger coverage area for disseminating safety-related information. Additionally, mechanisms for authentication and integrity validation are not supported.

A platform supporting a parking space finder application is presented in [17], based on an architecture for mobile devices and a web site for the registration of users and advertisements. This platform considers security aspects like the anonymity for users privacy by means of a zero-knowledge proof mechanism for granting nodes authentication, as well as an aggregation technique for signing alert messages in order to prevent sending false information. The experiments with this platform focus on traffic congestion and parking spaces detection, as well as parked vehicle finding and advertisement display. However, details about communications or security metrics are not specified, leaving the open issue of quantifying the overhead in time and space of the security scheme.

Lastly, the work in [18] proposes a VANET-based dissemination system for emergency vehicles. Among the defined design requirements, authors considered dissemination and security issues. This later one is addressed with a public key infrastructure (PKI)-based authentication mechanism, where digital signatures and their corresponding public key certificates are attached to each transmitted message. A prototype is deployed using personal computers on emergency vehicles, which is mainly validated in terms of how the system operates in real experiments. However, the impact of using the security service, namely certificates validation in PKI, on the dissemination process is not evaluated. Hence, it is not possible to determine the feasibility for deploying PKI approaches on mobile platforms for VANETs.

Table 1 summarizes the most representative approaches proposed in the literature for the deployment of smartphone-based VANETs and the extent to which security and multi-hop message dissemination has been considered. On the one hand, we can note from Table 1 that multi-hop message dissemination has not been widely considered in the designs. However, since the transmission range of nodes in a VANET is quite limited, multi-hop message dissemination protocols are essential to extend coverage area and thus, to increase the amount of informed vehicles. In such protocols, it is decided whether or not a message should be retransmitted based on a given dissemination criterion (e.g., transmitted power, distance). Notice that inefficient message dissemination approaches could translate into a reduced number of informed vehicles about a given traffic situation if messages are lost or delayed.

On the other hand, the mechanism for granting the integrity of the transmitted messages and the authenticity of nodes has been set as essential for proper operation in such an environment. According to Table 1, security mechanisms for authentication and integrity have been covered mainly through a PKI-based approach, which supposes the existence of a complex setting along the road to support digital signatures by means of digital certificates. However, the PKI approach is far from realistic in practical deployments of VANETs [21]. The most practical realization of VANETs in the short-term is based on inter-vehicle (V2V) communication enabled by smartphones for example, where infrastructure is generally not available and the existence of a PKI could not be guaranteed. As a more feasible alternative, digital signatures to guarantee authentication and integrity of messages in V2V could be better approached with a certificateless signature scheme (CSS) well suited for infrastructure-less VANET scenarios.

### 1.3. Contributions

Taking into account the aforementioned context, this work provides the following contributions.

- A methodological approach for the design of a mobile platform for enabling the provision of a novel certificateless security approach in a smartphone-based VANETs. In particular, the platform is intended to disseminate sensitive information and as such authentication and integrity checks are mandatory in our design.
- The construction in the asymmetric setting of a Certificateless Signature Scheme (ACSS), relying on pairing-based cryptographic signatures, that ensures authenticity of nodes and integrity of exchanged data among smartphones in VANETs. We present a novel construction of digital signing algorithm based on pairing-based cryptography under the asymmetric setting. The asymmetric property allows practical implementations for security levels compliant with current standards (e.g., 128-bits or greater).
- For the first time, a proof-of-concept, deployment of the ACSS scheme in a smartphone-based platform in VANETs is presented in the literature. We validate our platform over small-scale deployments, using as a baseline a multi-hop message dissemination protocol. Nevertheless, the platform can be used to deploy other message dissemination protocol as required.

The rest of this paper is organized as follows. Section 2 presents an overview of security aspects related to VANETs. In Section 3 the design and main building blocks of the proposed platform are described. Section 4 presents the proposed certificateless digital signing approach. In Section 5 the implementation process of the platform in Android-powered devices is detailed. Then, Section 6 presents the methodology and results of the experiments to validate the proposed smartphone-based platform. Finally, Section 7 concludes this work.

## 2. Security in Vehicular Networking

Security services in VANETs are of paramount importance, mainly because of the inherent nature of the communication channel where any malicious user could manipulate a given message leading to a malfunction of the network or to compromising the operation of safety-related applications [22,23]. Notice that the manipulation of life-critical information (e.g., from satefy-applications) in VANETs could lead to life-threatening situations. From a message-centered security viewpoint, attacks in VANETs by a malicious node (internal or external) include: record messages and later injection (Replay), message modification before forwarding (Change), message destruction after reception (Delete), and creation of fake messages and their injection in the network (Manufacture). The attacker could mislead other vehicles or impersonate road side units (RSU) to spoof false service advertisements or safety hazard warnings. Thus, securing the dissemination process in VANETs is crucial [22].

Most of the known attacks in VANETs are likely to be executed by an insider attacker, a malicious node that does not follow the protocol or that impersonates legitimate nodes, and transmitting false information to contaminate the communication network [24,25]. To overcome common attacks, the IEEE 1609.2 working group (https://standards.ieee.org/findstds/standard/1609.2-2016.html) recommends to implement digital signatures by means of PKI and particularly, Elliptic Curve Cryptography [26] to ensure authentication and integrity of the data interchanged by vehicles. The PKI is responsible for creating, distributing, and managing the public/private keys of vehicles in the VANET by means of digital certificates. A signed message will contain a signature that can only be generated by some private key that the sending node (vehicle) possesses, while allowing any node receiving the signed message to be able to verify the signature with public key included in the message (mathematically related to the secret key).

It is worth noting, however, that the PKI approach is far from realistic in practical deployments of VANETs due to the high cost implied to install RSU [21]. Therefore, digital signatures without relying on a PKI are needed to guarantee authentication and integrity of messages in V2V scenarios. In this regard, a CSS approach for VANETs security in V2V communications is proposed in [27], assuming that road-side infrastructures are not available. The CSS realization in that work avoid the inherent key escrow problem of identity-based signature. Furthermore, the CSS scheme [27] considers the interaction of vehicles equipped with OBUs and RSUs, as well as a Trusted Authority (TA). The vehicles participating in the scheme must be enrolled and the cryptographic material (keys) is generated and distributed by the TA. Once the scheme is prepared and vehicles adhere to a VANET, each one is able to generate the signature for a warning message previous to its transmission and to verify the signature of a received message. This way, nodes in the VANET can guarantee that the exchanged messages have not been modified and hence to legitimate the source. However, the CSS approach, as presented in [27], is defined over cryptographic pairings limited to the symmetric setting. Symmetric pairings have the disadvantage that they are only viable for practical implementations using obsolete security levels (for example, 80-bit) [28].

## 3. Smartphone-Based Platform Design

Taking into consideration the previous analysis, we present a smartphone-based platform based on two main pillars, namely security provision and multi-hop message dissemination for V2V scenarios. Unlike existing work (see Table 1), we consider these two aspects pivotal to develop an integrated platform to allow the design, deployment, and evaluation of secure message dissemination approaches in V2V experimental scenarios.

### 3.1. Platform Requirements

In this work, the smartphone-based platform design is driven by the following functional and non-functional requirements (FR and NFR, respectively).

- FR1: Message dissemination. The platform must behave either as a source node that starts the transmission of a message or as a relay that rebroadcasts a received message whenever a dissemination criterion is fulfilled.
- FR2: Security. The platform must provide authentication and integrity security services in a per-packet basis.
- FR3: Location-awareness. The platform must retrieve the current location of the device by means of a native location provider or any Bluetooth-enabled GPS device. While the first option is generally adopted, because the convenience and ease of use, the second alternative can be helpful in experiments aimed to contrast precision of location information from different providers. It should also be able to adjust intervals for location requests, which could be useful for deploying energy- and context-aware oriented mechanisms in VANET mechanisms and applications.
- FR4: Performance metrics. The platform must monitor and store relevant metrics related to message dissemination (e.g., delay, losses) and security services (e.g., execution time, memory usage). The collected metrics should be stored locally, and global statistics should be accessible by the user.
- FR5: Packet management. The platform must support a flexible packet management mechanism to enable the definition of application specific packet formats.
- NFR1: Passiveness. The user should not be aware of the underlying operation mechanisms on the smartphone-based platform for secure message dissemination. Moreover, minimal user involvement should be required for the configuration of basic elements.
- NFR2: Modifiability. The platform components should be organized to ease the understanding of its functional principles and interaction among components, allowing the integration of new functionalities.
- NFR3: Energy. The platform should provide the means for deploying mechanisms to drive a trade-off between energy efficiency and data transmission.
- NFR4: Network management. A given node running the platform should create, discover, and connect to the VANET without the intervention of any managing entity or infrastructure.

### 3.2. Platform Modules Definition

**Figure 1:**
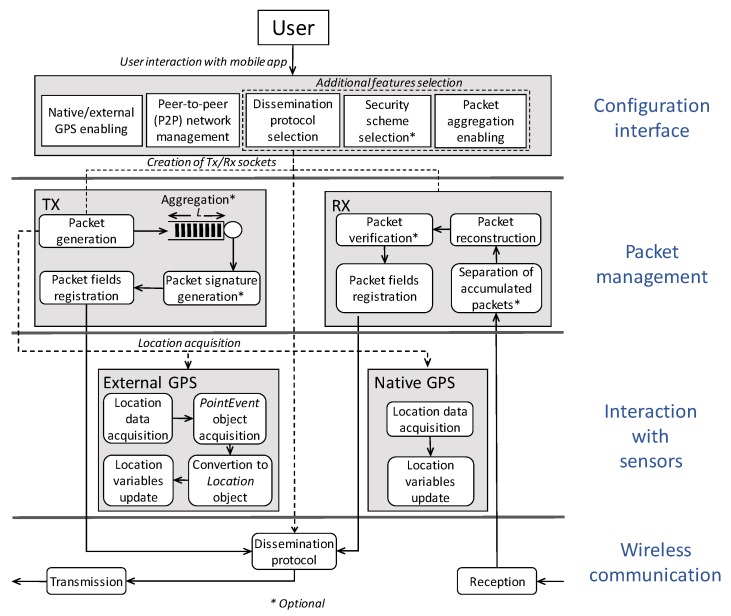
Building blocks of the smartphone-based platform for secure message dissemination. The platform is intended to support both the transmission (TX) and reception (RX) roles to enable a given node behave as source for packet generation, relay for disseminating messages, or as destination node. Specific functions (marked with *) at the TX/RX modules are optional, and should be enabled at the user space (i.e., configuration interface).

Figure 1 illustrates the modules of the proposed smartphone-based platform for secure message dissemination in VANETs. The smartphone-based platform provides a user space layer to configure the core security and dissemination parameters being taken into account in a given experimental V2V scenario. More specifically, such settings are performed to configure the GPS provider for location updates, to select the statistics of interest to be recorded, and to define security parameters. A practical interpretation of the smartphone-based platform operation is as follows.

- Once the initial parameters have been introduced, the platform firstly creates the required sockets for message transmission and reception. This is because in our design a smart device deploying the platform could behave either as a source or as a relay node. A source node is the one that starts broadcasting a warning message to inform about a given event inferred from its sensed context. A relay node is expected to extend coverage transmission by rebroadcasting an incoming message. Although in this case no additional payload would be added to the received message, the node would require its contextual information (e.g., location, power, etc.) to determine if the considered dissemination criterion is fulfilled.
- At the application framework layer, the packet management module provides the functionality of packet generation according to a predefined packet format as illustrated in Table 2. Although the packet format could be easily modified to prevent large overhead amounts, the packet management component could also support power-aware strategies to drive trade-off between transmission packet delay and overhead-payload ratio. Each generated packet triggers an event at the storage module that records outgoing/ingoing packets processed by the platform.
- The wireless interface manager location at the application framework layer aims to control the interaction with the location providers. Unlike existing works that mainly rely on native GPS receiver, depending on energy constraints and location accuracy, our platform is designed to acquire location data from a native GPS sensor or an external GPS logger device accessed via Bluetooth. Regardless of the location provider, when the node deploying the platform needs to disseminate a message, at the TX module the location information is appended on each generated packet. On the contrary, when a message is received (RX module) it is verified and registered and then it is passed to the dissemination protocol module. This latter one decides whether the received message should be retransmitted to the next hop or not, taking into account a distance criterion.
- Upon the transmission or reception of a packet, a record of its payload is saved in a file. The information stored in the file conforms the Packet fields registry, which is maintained in the main storage directory of the device. The file (i.e., registry) contains the data of each packet that is part of an existing communication. A text string containing the payload’s fields is obtained from the object modeling the packet. The resulting file is available for statistical analysis offline at the end of the test trials.

### 3.3. Security Services in the VANET Platform

The smartphone-based VANET proposed in this work uses digital signatures to guarantee the authentication and integrity services in message dissemination. As argued before, our digital signature scheme does not follow the IEEE 1609.2 recommendation. The main reason is that the IEEE 1609.2 standard assumes the existence of an infrastructure for deploying a PKI-based security solution for secure message exchange in VANETs [29,30,31]. In this context, we demonstrate that digital signatures without relying on a PKI are possible to deploy in a smartphone-based solution as the one proposed in our work. We follow that approach and extend the CSS scheme proposed by Malip et al. [27] to construct a novel certificateless scheme, referred to as ACSS (to highlight the asymmetric property), which is intended to provide integrity and authentication services for messages disseminated in the smartphone-based VANET proposed in this paper. The proposed ACSS is based on the generation of the necessary keys and related material for the signature and verification operations in an offline stage. Global parameters are generated by a Trusted Authority or TA (car manufacturer, vehicle registration office, etc.). Later, in an online stage, the scheme allows signature and verification operations over the messages exchanged by the nodes (vehicles) of a VANET. Details of the novel ACSS scheme are given in the next section.

## 4. Asymmetric Certificateless Signature Scheme (ACSS)

In this section we firstly provide background information regarding certificateless public key cryptography, focusing on the existing CSS scheme [27]. Then, we detail the proposed ACSS to provide integrity and authentication secure message dissemination in the proposed smartphone-based VANET. Specifically, we provide details of a new construction for certificateless digital signatures based on the CSS scheme reported in [27], but realized in the asymmetric setting, which enables using different (higher) security levels and explore performance-security tradeoffs.

### 4.1. Preliminaries

**Figure 2:**
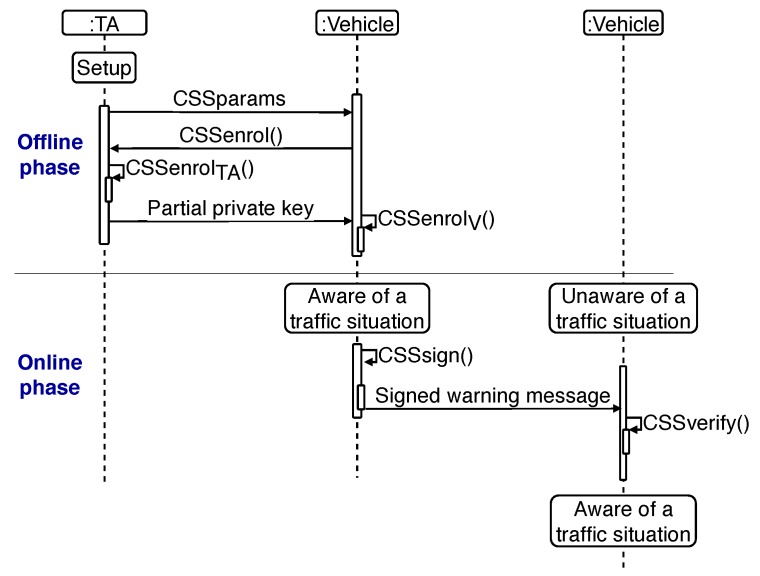
Sequence diagram of the certificateless signature scheme (CSS) scheme.

Certificateless public key cryptography [32] is a model intermediate between traditional PKI and Identity-based cryptography (IBE) [33], where an identity public value (vehicle identification number, license plate number, etc.) serves as public key. In this sense, Figure 2 illustrates the CSS scheme [27] and the interaction among its actors.

The CSS scheme operation consists of two phases. The first one (offline) involves the TA running the *setup()* procedure for a security level *k*. This procedure generates the system parameters (*params*) and partial private keys, which are distributed offline to all the nodes (vehicles). A vehicle must execute the *enroll()* procedure with the TA, sending public values. With this information, the TA executes a local computation (*enroll_TA_()*) in order to generate and send (securely) to the vehicle a private key. With this information, the vehicle executes a local computation (*enroll_v_()*) to generate its final credentials and material for secure communication in the VANET.

The second phase occurs during message exchange in the VANET (online). With the material generated in the offline phase, vehicles will be able to sign (*sign()*) and verify (*verify()*) the outgoing or incoming messages, respectively.

The original CSS scheme [27] is constructed over symmetric bilinear pairings defined over groups. Pairings computation constitutes the critical operation that strongly affects both the efficiency and security of all pairing-based cryptographic schemes [34], including the CSS scheme. Let G and $G_{T}$ be cyclic groups of prime order *r* [35]. A bilinear symmetric pairing or bilinear mapping is an efficient computable function $e:G\times G\to G_{T}$, such that

1. $\forall a,b\in Z_{r},e\left(g^{a},g^{b}\right)=e{\left(g,g\right)}^{ab}$, with *g* a generator in G.
2. e(g,g)≠1

In a group G with generator *g* and order *r*, $y=g^{a}$ means to accumulatively apply the group operation to *g*, a−1 times, having y∈G again, for any positive integer *a*. From this, the discrete logarithm problem states that given *g* and $g^{a}$, for enough large order *r*, it is infeasible to compute *a* [35]. Under this problem, a public key cryptosystem can be built, using $\left\{a,g^{a}\right\}$ as a key pair for encryption or digital signatures: *a* is the private key and $g^{a}$ is the public one.

Although the CSS scheme as proposed in [27] is recommended for VANETs security, its definition in the symmetric setting limits its applicability in real scenarios such as smartphones and other smart devices. According to practical realizations of pairing-based encryption [28,36,37], the symmetric pairing is realized by using type *A* elliptic curves, only efficient for obsolete security levels (80-bit security level or less) according to standards such as NIST [38]. Currently, the recommended security levels are for 112, 128, 192, or 256 bits.

A way to use increased security levels in pairing-based cryptography is to use the asymmetric setting, usually realized with type *F* elliptic curves. In [28], the asymmetric setting for pairings was proposed and evaluated in the context of data security in cloud storage. The results achieved showed the efficiency of using asymmetric pairings, in terms of higher security and lower overhead in running time and memory requirements. Thus, we re-define the original CSS scheme and provide a new construction named ACSS in the asymmetric setting.

### 4.2. Description of Our ACSS

Details of the ACSS scheme are given hereafter. A bilinear pairing in the asymmetric setting is defined as follows: Let $G_{1}$, $G_{2}$, and $G_{T}$ be cyclic groups of prime order *r*, with $G_{1}$ different to $G_{2}$. The asymmetric bilinear pairing is the efficient computable function $e:G_{1}\times G_{2}\to G_{T}$, such that

1. $\forall a,b\in Z_{r},g_{1}\in G_{1},g_{2}\in G_{2},e\left(g_{1}^{a},g_{2}^{b}\right)=e{\left(g_{1},g_{2}\right)}^{ab}$
2. $e(g_{1},g_{2})\neq 1$

$G_{1},G_{2}$ are subsets of elliptic curve groups defining an additive group. The asymmetric pairing can be transformed in the symmetric one by taking $G_{1}=G_{2}$ and $g_{1}=g_{2}$, however, the opposite is not straightforward.

The new ACSS scheme is composed of the procedures *ACCSSsetup()*, *ACSSenrol()*, *ACSSenrol()*, *ACSSsign()*, and *ACSSverify()*, which are defined in the following.

The *ACSSsetup()* procedure creates *e* for enough large groups $G_{1}$, $G_{2}$, $G_{T}$, and $Z_{r}$, so the discrete logarithm problem be hard to compute. From $G_{1}$ and $G_{2}$, generators $g_{1}$ and $g_{2}$ are selected. Three cryptographic hash functions $H_{1},H_{2},H_{3}$ are selected, each one mapping from ${\left\{0,1\right\}}^{*}$ to $G_{1}$. An integer *s* is uniformly selected at random from the set $Z_{r}^{*}$ and used as the system *master secret key*. Finally, the setup procedure computes $g_{\mathrm{pub}}={\left(g_{2}\right)}^{s}$ as the *system master public key*. The *ACSSsetup()* procedure outputs [*s*, *ACSSparams* $=<e,r,g_{1},g_{2},g_{\mathrm{pub}},H_{1},H_{2},H_{3}>]$. The master secret key is kept confidential whereas the tuple *ACSSparams* is published as the public system parameters, shared by all participants.

The enrollment algorithm is executed by both the TA and the vehicle *v* to be enrolled. Thus, this protocol consists of two parts: *ACSSenroll_TA_(ID_v_)*, where $ID_{v}\in {\left\{0,1\right\}}^{*}$ is the vehicle unique identifier and *ACSSenroll_v_(x_v_)*, where $x_{v}$ is the vehicle partial private key generated by the TA. The *ACSSenroll_TA_(ID_v_)* procedure is executed as follows:

- $ID_{v}$ is the vehicle unique identifier (e.g., license plate).
- Compute $h_{1}$ = $H_{1}\left({\mathrm{ID}}_{v}\right)$ (in $G_{1}$).
- Compute $x_{v}$ = ${\left(h_{1}\right)}^{s}$. $x_{v}$ is the partial private key for the vehicle *v* (in $G_{1}$).

The partial private key $x_{v}$ is actually a signature on the vehicle ID, and the vehicle *v* can check its correctness by checking whether $e\left(x_{v},g_{2}\right)=e\left(h_{1},g_{\mathrm{pub}}\right)$.

By the side of the vehicle, the *ACSSenroll_v_(x_v_)* procedure is as follows:

- $x_{v}$ is the vehicle partial private key.
- Choose a random value *a* in $Z_{r}^{*}$ (the set {1,2,…,r−1}).
- Assign the tuple $\left\{x_{v},a\right\}$ as the private key of *v*.
- Assign the value ${\left(g_{2}\right)}^{a}$ as the public key of *v*.

**Figure 3:**
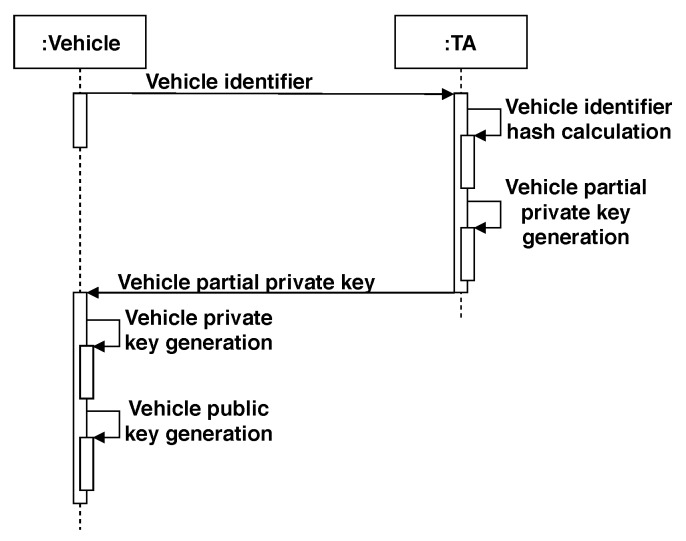
Sequence diagram of the enrollment process in the CSS (and asymmetric CSS [ACSS]) scheme.

A summary of the messages and actions performed by the vehicle and the TA during the enrollment phase is depicted in Figure 3.

Finally, in the second phase of the scheme only the vehicles are involved, performing the signature generation and signature verification operations. Vehicles inform others about a traffic situation, mainly through warning messages dissemination. These messages must be signed by the transmitter, then a receiver of the message verifies its signature. For signing, a vehicle executes the following operations:

- *M* is the message to be signed by *v*.
- ${\mathrm{ID}}_{v}$ is *v*’s identifier.
- ${\mathrm{SK}}_{v}=\left\{x_{v},y_{v}\right\}$ is *v*’s private key.
- ${\mathrm{pk}}_{v}$ is *v*’s public key.
- *v* chooses a random value *a* in $Z_{r}^{*}$ (the set {1,2,…,r−1}).
- *v* computes $u={\left(g_{2}\right)}^{a}$ (in $G_{2}$).
- *v* computes $h_{1}=H_{2}\left(M,{\mathrm{ID}}_{v},{\mathrm{pk}}_{v},u\right)$
- Compute $h_{2}=H_{3}\left(M,{\mathrm{ID}}_{v},{\mathrm{pk}}_{v}\right)$
- Compute $t=x_{v}+{\left(h_{1}\right)}^{a}+{\left(h_{2}\right)}^{y_{v}}$, where ’+’ is the group operation in $G_{1}$.
- The digital signature of *M* by *v* is $\sigma {\left(M\right)}_{v}=\left(t,u\right)$.

A vehicle $v_{2}$ receiving the signature $\sigma {\left(M\right)}_{v}$, $v^{\prime }s$ public key, and *v*’s ID will be able to verify the authenticity and integrity of *M* by executing the following steps:

- *M* is the received message and its signature is $\sigma {\left(M\right)}_{v}=\left(t,u\right)$.
- $ID_{v}$ is *v*’s identifier.
- $pk_{v}$ is *v*’s public key.
- Compute $h_{1}=H_{1}\left({\mathrm{ID}}_{v}\right)$ (in $G_{1}$).
- Compute $h_{2}=H_{2}\left(M,{\mathrm{ID}}_{v},{\mathrm{pk}}_{v},u\right)$ (in $G_{1}$).
- Compute $h_{3}=H_{3}\left(M,{\mathrm{ID}}_{v},{\mathrm{pk}}_{v},\right)$ (in $G_{1}$).
- If $e(t,g_{2})$ equals to $e\left(h_{1},g_{\mathrm{pub}}\right)\times e\left(h_{2},u\right)\times e\left(h_{3},{\mathrm{pk}}_{v}\right)$, the signature is valid and the message is considered authentic. If not, the message should be discarded. Here, the ’×’ operator is the group operation in $G_{T}$.

The correctness of the above scheme can be verified from the fact that $x_{v}={\left(h_{1}\right)}^{s}$. That is:

(1) $$\begin{array}{cc} e(t,g_{2}) & =e\left({\left(h_{1}\right)}^{s},g_{2}\right)\times e\left({\left(h_{2}\right)}^{a},g_{2}\right)\times e\left({\left(h_{3}\right)}^{y_{v}},g_{2}\right)\\ \; & =e\left(h_{1},{\left(g_{2}\right)}^{s}\right)\times e\left(h_{2},{\left(g_{2}\right)}^{a}\right)\times e\left(h_{3},{\left(g_{2}\right)}^{y_{v}}\right)\\ \; & =e\left(h_{1},{\left(g_{pub}\right)}^{s}\right)\times e\left(h_{2},u\right)\times e\left(h_{3},pk_{v}\right) \end{array}$$

**Figure 4:**
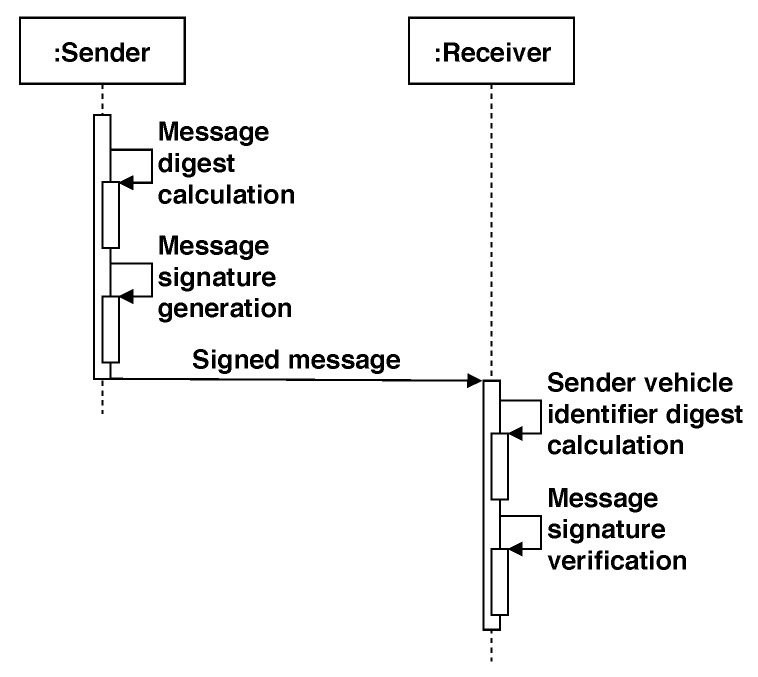
Sequence diagram of the Sign-Verify process in the CSS (and ACSS) scheme.

Figure 4 summarizes the signature generation and verification processes between two participant vehicles.

### 4.3. Security Assumptions

The ACSS scheme proposed in the previous section was verified to be correct. In order for the scheme to be secure, the following assumptions must be met:

- The order *r* for the groups $G_{1}$, $G_{2}$, and $G_{T}$ must be compliant with recommended values in standards (i.e., recommended security levels in [38]). The security settings for the asymmetric pairing provided in [28] are a reference point.
- The hash functions $H_{1},H_{2}$, and $H_{3}$ must be secure, collision free. Algorithms for mapping bit-strings to groups showed to be secure must be used, for example [36,37].
- The master private key must be kept secure and the transport of partial private keys from the TA to the vehicles during the enrollment process must be secure, for example, using a SSL-enabled connection. Another alternative is the car manufacturer to install the key material in the vehicle offline.

## 5. Android Implementation

The smartphone-based VANET solution was implemented as a middleware for the Android platform, which provides a flexible development environment for accessing and controlling sensors’ information. However, the design principles defined in Section 3 are valid for experimenting with other mobile platforms, which is out of the scope of this work. In the following, we describe the deployment of the proposed solution on the Android software stack.

### 5.1. Wi-Fi Peer-to-Peer (P2P)

The message dissemination is performed between smartphones without the need of a network infrastructure, using the Wi-Fi P2P APIs for Android. This enable smartphones to discover nearby available peers (mobile devices) and to establish connectivity for information exchange. We also use the Wi-Fi P2P Manager class, which returns a channel that connects our VANET platform to the Wi-Fi P2P framework. This makes it possible to initiate the discovery of available peers to start the connection using a set of available function calls for managing a list of neighboring mobile devices. Moreover, we set up a BroadcastReceiver to listen to broadcast intents and to manage notifications regarding the connection state, new available devices, disconnections, etc. In particular, the notifications of default methods of the Wi-Fi P2P Manager class are managed by the BroadcastReceiver.

In the deployed P2P scheme, each smartphone is able to act as a server listening for new received messages, or as a client generating and transmitting messages to another device. In order to properly manage such communication roles in our VANET platform, the following classes were implemented:

- The Server class, to create objects that listen on a port for newly received messages. This object keeps listening in the same port for new messages, and dispatch each one in a separated thread.
- The ServerTasks extends from the AsyncTask class and operates in the background. This class processes every received packet and obtains the reception timestamp. In addition, the message content fields are then stored in a file for future analysis and statistics extraction.
- The Client class has the main function of sending packets and appending them relevant data, such as GPS position and a unique identifier. This class also extends from AsyncTask, with the purpose of performing its operations in the background.

### 5.2. Packet Management

The information exchange between the client and server classes is assumed to be based on a per-packet basis. A packet is composed by a payload and its corresponding overhead. We implement the Packet class as a serializable interface to allow both, the transfer of a complete object and working at byte level during the transfer. As defined in Table 2, the payload of each packet contains information related to the message type to be disseminated among nodes. It also includes the following fields: identifier, MAC address, and IP address of the device, timestamp of packet creation, latitude, longitude, and speed of the node. The packet uses a field to indicate whether or not security services are enabled (i.e., message signature is appended or not). Moreover, the overhead of each packet is defined in terms of the amount of data required to provide security services to each packet (message signature).

In the MAC address, the 1st to 3rd octets define the Organisationally Unique Identifier (OUI), whereas the 4th to 6th octets correspond to the Network Interface Controller (NIC) Specific. In order to provide uniqueness to generated packets, the three octets of the NIC from the device’s MAC address and the system’s time (in nsec) are used as input to a hash function. From the resulting digest, we use the first seven characters as the packet identifier.

#### 5.2.1. Packet Aggregation Mechanism

Energy-efficient data transmissions are crucial in energy resource constraints devices like smartphones. Thus, we define a packet aggregation mechanism aimed to control the payload and overhead ratio. This mechanism reduces, to some extent, the impact of data transfers on the energy consumed by the devices at the expense of an increased packet latency. The trade-off between latency and aggregation level is application specific, and can be managed in the aggregation mechanism by tuning the queue length (*L*) (see Figure 1). Hence, the proposed mechanism aggregates *N* ≤ *L* packets before the signature’s generation and transmission process occur. For this, it is required to get the bytes of each packet object to concatenate it. Once *N* is reached, the resulting aggregated message is passed to the signature operation and then transmitted.

#### 5.2.2. Packet Records

The packets that are transmitted or received are registered into files saved in the device’s local storage. Each file contains a row for each packet along its fields and the corresponding timestamps of generation and reception nodes. The Java OutputStreamWriter and the BufferedWriter classes are used to create the file and to write on the output file, respectively. Hence, we obtain a string with the payload of the object that models the packet in our smartphone-based VANET platform.

### 5.3. GPS Location Readings

A core requirement in our proposal is to provide location-awareness. The location updates are accessed using the LocationManager class provided in the Android API. This class provides the getLastKnownLocation method that returns a Location object with the location information. The method requestLocationUpdates of the class LocationManager is invoked to start the reading acquisition process and a Listener is used to update the values of latitude, longitude, and speed each time a location event occurs.

The location readings in the platform deployed in Android could be either acquired from the native GPS embedded in the mobile device or from an external GPS receiver connected to the smartphone via Bluetooth. We explored how an alternative location provider with high location data granularity could impact the behavior of message dissemination in VANETs. To this end, an external GPS receiver can be linked to the device through a Bluetooth connection from high resolution location updates. The location updates from an external GPS receiver can be managed from its known universal identifier (UUID). Such updates are processed by the platform by converting from the source format (NMEA) to geographic coordinates (latitude, longitude) to then keep them available for the rest of the modules.

### 5.4. The Security Scheme

The deployment of the ACSS scheme in each node was done using the jPBC library [37]. jPBC allows performing the mathematical operations underlying the ACSS scheme directly in Java. The elliptic curve and asymmetric pairing parameters for different security levels were taken from the settings previously reported by Morales et al. [28].

At the beginning, all the nodes to be participant in the VANET execute the offline phase, to ensure the cryptographic keys and related parameters in the ACSS scheme for a given security level are properly established in advance.

## 6. Experimental Results

In this section we describe the experimental settings and the obtained results aimed to validate the proposed smartphone-based VANET platform. To this end, we concentrate on the following aspects:

- *Message dissemination evaluation.* The aim of these experiments is to validate the platform’s feasibility for continuous packet generation, transmission, and reception in multi-hop conditions. Then, we also evaluate the performance of a location-based message dissemination protocol to determine the influence of the location provider’s precision on the message dissemination process for selecting the next relay node.
- *Security services.* We analyze the impact of deploying the proposed ACSS security scheme on our smartphone-based VANET platform in terms of delay and packet loss under different security levels.
- *Packet aggregation.* We determine the benefits of deploying packet aggregation mechanisms.

In the outdoor experiments, we deployed the proposed platform in three Android-powered devices: two Samsumg Galaxy Note 10.1 tablets and one Samsung Galaxy Grand Prime smarpthone. The main technical specifications of these devices are summarized in Table 3. We also use three Qstarz BT-Q1000eX GPS logger units in the experiments for acting as external GPS receiver connected to each mobile device via Bluetooth. The GPS loggers provide GPS fixes with a sampling frequency of 10 Hz, allowing us to contrast the impact of more accurate GPS fixes on the message dissemination process. Additionally, in the offline phase performed for tuning the security services, we use a laptop Lenovo Thinkpad Edge 14 which acts as the Trusted Authority in the security scheme.

As explained in Section 3, the initial parameters should be properly defined through the configuration interface before each experimental trial. This is the case when selecting the GPS provider, dissemination criterion, security scheme, and aggregation technique. It is worth noting that we assume that inter-vehicle communication takes place by means of the mobile devices running our platform. However, the link between the proposed platform and its usability in vehicles for enabling intra-vehicle communication is not analyzed in our experiments.

### 6.1. Message Dissemination Evaluation

We firstly consider a scenario were the two Samsung tablets have been used for message transmission-reception in a single-hop scenario. The distance between devices was set to d={10,20,50,100} m. For each case, one device was configured to continuously generate and transmit dummy packets with an interarrival packet time of 5 s during a period of around 5 h (3720 packets were sent). Then, we analyze the behavior of the transmissions in terms of the Round Trip Time (RTT) and packet loss metrics. The RTT delay measures the elapsed time between packet’s transmission and the reception of its respective ACK packet. The RTT metric was preferred over the delay in one hop to prevent any biasing factor related to the devices’s clocks (i.e., inaccuracies due to synchronization errors). Table 4 shows a trend of increased values as the separation distance of the devices increases, for both observed metrics. This is an expected behavior because of the power signal reduction (attenuation) during transmissions as the travel distance increases.

The previous experiment allows us to validate the platform’s feasibility in simple single-hop conditions. We define the feasibility as the ability of the platform for continuous packet generation, transmission, and reception in the mobile devices without premature finalization caused by CPU and memory consumption usage issues.

**Figure 5:**
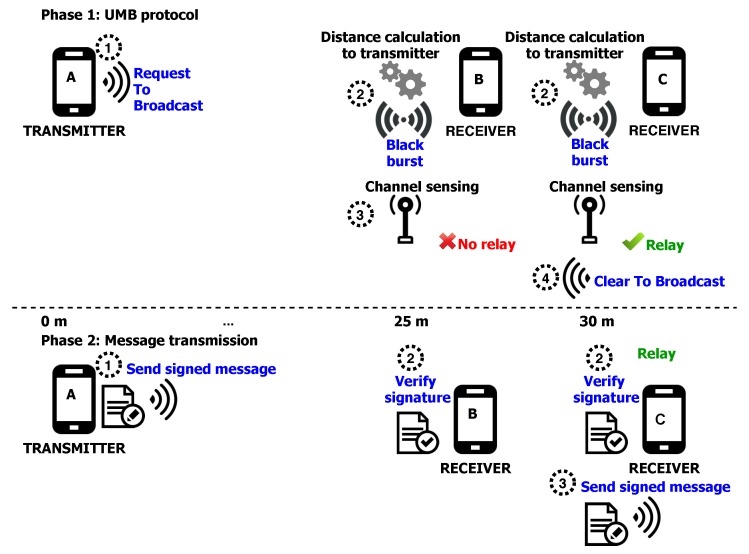
Relay node identification (**phase 1**) and transmissions behavior (**phase 2**) with the Urban Multi-hop Broadcast (UMB) message dissemination protocol.

The second experiment is aimed at validating the message dissemination functionality of our platform in practical trials. Taking into account that distance between nodes has become the “de facto” standard in the design of broadcast dissemination protocols in VANET, we select the Urban Multi-hop Broadcast (UMB) protocol, proposed by Korkmaz, et al. [39], which is a location-based protocol. Note that the modular design of the platform makes possible the deployment of other message dissemination protocol as required. Figure 5 illustrates the protocol’s operation. Under the UMB protocol, when a node aims to transmit a new message, it must firstly send a Request To Broadcast (RTB) message containing its location. A node receiving the RTB then computes its distance to the sender. Then, the receiver transmits a Black Burst signal during a time period that is function of the transmitter-receiver distance. Once that time is reached, the receiver senses the channel looking for other nodes’ Black Burst signal. When the receiver does not detect any signal whatsoever, that implies that such receiver is the farthest away from the sender, so it becomes the retransmitter in charge of sending the next message beyond the coverage range of the original sender and it responds the sender with a Clear To Broadcast (CTB) message.

The devices used in the second experiment were a Samsung smartphone (node A), two Samsung tablets (nodes B and C), and three GPS logger units. Node A acts as the transmitter that continuously sends packets towards nodes B and C. The distance between the transmitter and each receiver is $d_{A-B}=25$ m and $d_{A-C}=30$ m. We assume no mobility. In order to evaluate the performance of the UMB protocol, we concentrate in four representative metrics:

- Successful reception ratio. Ratio of the total number of nodes and the number of nodes that correctly received the packets.
- Generated load per-broadcast packet. Ratio of the average of total transmitted bits and the total number of broadcast packets received.
- Relay error selection. The average times the protocol selects an erroneous relay node for broadcast purposes.
- Packet loss ratio. The percentage of packets lost per number of packets transmitted.

The results of this experiment are summarized in Table 5. The reception levels are acceptable, with values close to 100% successful, especially due to the reduced number of nodes used in the tests (only three receivers were used), so when one of them did not receive a message, the value for this metric was reduced considerably. On the other hand, the average dissemination speed has been quantified in the farthest node (retransmitter), which has been located 30 m away from the transmitter. A very close value was observed in all cases, since the delay factor variation was not significant. As expected, higher load values are observed in cases where security is considered at the cost of reduced reception ratio. That is, the packet loss is higher when the ACSS scheme is considered due to the increase in the size of the transmitted packets.

Additionally, metrics that allow evaluating the performance of the platform have been analyzed, namely, the error percentage that occurs in the selection of the relay node and the packet loss in different segments of the network. As it is graphically shown in Table 6, in the case where location readings of the external GPS were considered, a lower error rate was presented. This is attributed to the distance calculation that is required to get the time duration of the Black Burst transmission according to the UMB dissemination protocol. This indicates that the accuracy of the external GPS receiver has been proven to be higher than the one with native sensor. We also observe that packet loss presents the highest values in the transmission from node C to node A, followed by those from node A to node B, while those from node B to node C have the lowest values. This is attributed to the separation distance between nodes, 30, 25, and 5 m, respectively.

### 6.2. Impact of the Security Scheme

The implemented ACSS security scheme can perform bilinear pairings under the symmetric (using $G_{1}=G_{2}$) and asymmetric settings. Furthermore, the security level set for the overall scheme provides different configurations for a trade-off between higher security and time invested for the scheme operations. Table 7 presents the size for the groups involved in the security operations. Observe how the asymmetric setting uses groups of less size if compared with the symmetric one. This is an important aspect because the size of the signature attached in the messages exchanged by the vehicles is shorter.

The implications of the ACSS scheme for the platform are presented in the processing time for the signature generation and signature verification operations in addition to the tasks of the communication platform itself, resulting in an intra-nodal delay preceding the transmissions. Therefore, we have quantified the processing time for each operation in the platform varying the security level (80–128 bits) using the asymmetric bilinear pairing setting in the ACSS scheme. Table 8 and Table 9 present the obtained results for the transmitter and receiver, respectively. The signature generation process impacts the most on the transmitter (99% of the processing time), while the rest of the operations have a processing time almost negligible. This behavior is also observed in the signature verification operation at the receiver node. It is also shown that using a higher security level increases the time invested in the ACSS operations. Thus, the implications of a higher security level must be considered by the application in turn.

The results presented in Table 8 and Table 9 regarding the impact of the ACSS scheme in our proposed VANET realization are presented only as a reference. This is done because in the literature, such a scheme in the context of security of smartphone-based VANET has not been previously reported. In [18], the security approach is PKI-based, which is opposed to the one we propose to avoid the use of PKIs. Furthermore, that work did not measure the cost of cryptographic operations to provide authentication and integrity checks as we did in our work. This is the same case for the works [17,19] that provide a kind of authentication security service. In [19] for example, a PKI approach is followed and the only timing results presented are for data transfers from the smartphone to the cloud provider, cloud server data processing, and data transferred form the cloud server to the smartphone, but no specific timing results are presented for the security services. In [17], the authors presumably had provided the authentication security service through the use of digital signatures and certificates, however there are no details on how those signatures are applied in the data flow, nor implementation results about the cost of security operations.

### 6.3. Packet Aggregation Evaluation

**Figure 6:**
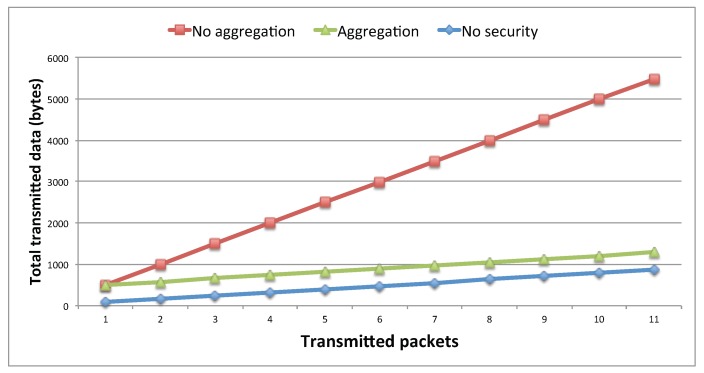
Total transmitted data for using and not using the aggregation technique.

The deployment of the security scheme comes at the cost of increased data flowing along the network due to the size of packets. Therefore, we implemented a data management mechanism for improving the smartphone battery usage by reducing the overhead to payload ratio. For this, we have implemented a data aggregation technique which can be useful for delay tolerant applications. This technique consists of the accumulation of *N* new generated packets in a buffer of length *L*. When *N* is reached, the accumulated data buffer is signed (ACSS scheme) and then transmitted. This way, only one signature is required for *N* accumulated packets, relieving the node from processing signature and verification operations for every packet and reducing the amount of data flowing though the network. Figure 6 shows that without any aggregation technique the data generated used is significantly higher. In fact, with the use of the aggregation technique, the data generated is close to the case without security scheme.

## 7. Concluding Remarks

In this paper, we have presented the design, development, and validation of a novel smartphone-based platform for secure multi-hop message dissemination in VANETs. For evaluation purposes, we have narrowed down the experimental trials to mainly validate the impact of the proposed ACSS scheme on the performance of the message dissemination process. In this sense, this paper has demonstrated that certificateless cryptographic schemes are a potential solution for ensuring data integrity and nodes’ authentication in VANETs. The platform supports the deployment of energy-efficient mechanisms to reduce, to some extent, the impact of the overhead introduced by the cryptographic schemes. In our experimental trial we used simple yet valid data aggregation-based and message dissemination approaches, which makes tractable the validation of the modules of the platform. However, more sophisticated energy efficient approaches and message dissemination protocols can be adopted in the platform due to its modular design and functional and non-functional requirements considered. The potential future work includes an exhaustive study about latency and energy consumption due to their importance in high priority messages. Similarly, we envision the evaluation of the key threats to security in VANETs by means of experimental trials with the proposed platform. We hope the ideas presented in this paper will encourage researchers and practitioners to develop more security-oriented approaches to enhance the reliability of VANET applications, which is an aspect that has remained almost unexplored in the area.

## Figures and Tables

**Table 1:** Smartphone-based vehicular ad-hoc network (VANET) platforms in the literature.

Year	Platform	Purpose	Disseminationn	Communication	Security Service
2017 [19]	Smartphones (Android)	Traffic event-based notifications	V2I (single-hop)	3G	Authentication
2016 [8]	Smartphones, tablets (Android)	Periodic message exchange	V2V (single-hop)	Wi-Fi	None
2016 [20]	Smartphones (Android)	Periodic message exchange	V2V (single-hop)	Wi-Fi	None
2016 [14]	Smartphones (Android), Raspberry Pi	Peer-to-peer video transmission	V2V (single-hop)	Wi-Fi	None
2013 [17]	Smartphones (Android)	Traffic event-based notifications	V2V (single-hop)	Wi-Fi	Authentication
2012 [15]	Smartphones (Android), Arduino	Traffic management	V2I (single-hop)	3G	None
2012 [16]	Smartphone (Android)	Periodic message exchange	V2V (multi-hop)	Wi-Fi Direct	None
2009 [18]	Laptops	Emergency unit warning messages	V2V, V2I (multi-hop)	Wi-Fi	Authentication
This work	Smartphones (Android)	Periodic message exchange	V2V (multi-hop)	Wi-Fi	Authentication, integrity

**Table 2:** Fields of the generated packets in the platform.

Field Name	Data Type	Size (bytes)
Packet identifier	String	7
Message type	Char	2
MAC address	String	17
IP address	Byte	4
Time stamp	String	23
Latitude	Double	8
Longitude	Double	8
Speed	Double	8
Security flag	Char	2

**Table 3:** Specifications of the devices used in the evaluation.

Feature/Device	Samsumg Galaxy Note 10.1	Samsumg Galaxy Grand Prime
System on Chip (SoC)	Exynos 4412	Snapdragon 410
CPU	Quad-core 1.4 GHz Cortex-A9	Quad-core 1.2 GHz Cortex-A53
GPU	Mali-400	Mali-400
Memory	2 GB RAM	1 GB RAM
Internal storage	64 GB	8 GB
Battery capacity	7000 mAh	2600 mAh
Android OS	Ice Cream Sandwich (4.0.3)	KitKat (4.4.4)
Wi-Fi	802.11 a/b/g/n, Wi-Fi Direct,	802.11 a/b/g/n, Wi-Fi Direct
Bluetooth	4.0	4.0

**Table 4:** Round time trip (RTT) and packet loss.

Distance (m)	RTT (ms)	Packet Loss (%)
10	106.56	33.89
20	111.15	27.14
50	112.55	45.65
100	114.92	48.12

**Table 5:** Successful reception ratio (Rx Ratio) and load generated per packet (Gen. Load) for the UMB protocol, with and without enabling security services in the platform.

Security	GPS	Rx Ratio (%)	Gen. Load (bits)
Not enabled	Native	79.13	1754.49
	External	90.36	1722.13
Enabled	Native	57.94	10,264.78
	External	85.21	10,820.89

**Table 6:** Relay error selection and packet loss ratio for the UMB protocol, with and without enabling security services in the platform.

GPS Provider	Security	Relay Selection Error (%)	Packet Loss A–B (%)	Packet Loss B–C (%)	Packet Loss C–A (%)
Native	Not enabled	53.13	19.41	17.22	25.97
	Enabled	84.38	17.13	12.80	21.24
External	Not enabled	40.63	8.18	7.42	13.32
	Enabled	65.63	6.12	4.78	33.46

**Table 7:** Fields elements size (in bits) in different configurations for the ACSS scheme.

Sec. Level/Groups Size	Sym			Asym		
	$G_{1}$	$G_{2}$	$G_{T}$	$G_{1}$	$G_{2}$	$G_{T}$
80	1024	1024	1024	320	640	192
112	2048	2048	2048	448	896	2688
128	3072	3072	3072	512	1024	3072

**Table 8:** Processing time (ms) for transmitter operations.

Operations	Security Level (bits)		
	80	112	128
Message generation	5.22	4.88	5.00
Parsing to bytes	0.65	0.53	0.52
Signature regeneration	1231.01	1628.67	1894.17
Output to interface	0.53	0.51	0.54
Other	2.85	2.70	3.15
Total	1240.26	1637.29	1903.38

**Table 9:** Processing time (ms) for receiver operations.

Operations	Security Level (bits)		
	80	112	128
Message reconstruction	7.18	6.62	8.08
Signature regeneration	329.07	443.71	453.48
Other	0.03	0.03	0.03
Total	336.28	450.36	461.59
